# Supplementary material for: Facile Fabrication of Diatomite-Supported ZIF-8 Composite for Solid-Phase Extraction of Benzodiazepines in Urine Samples Prior to High-Performance Liquid Chromatography
Source: Molecules. 2021 Aug 27;26(17):5209. doi: 10.3390/molecules26175209 (PMC8434582; doi:10.3390/molecules26175209)
Supplement: Supplementary file 1 [file molecules-26-05209-s001.zip › molecules-1326746-supplementary.pdf]

## Optimization of SPE conditions

Four washing solution of $NH_4OAc$ (25 mmol/L, pH=5), $NaH_2PO_4$ (25 mmol/L, pH=5), $H_2O$ and ACN/$H_2O$ (95/5, v/v) were studied with 8 mL of diluted urine (urine/$H_2O$, 3/1, v/v). $NaH_2PO_4$ (25 mmol/L, pH=5) gives the best recovery of 98.1±1.2%-100±2.7% (Figure S1a).

Three elution solvents including ACN, acetone and MeOH and elution volume (2, 3, 4 and 5 mL) were optimized. Figure S1b and c illustrate that 4 mL of MeOH exhibits the best extraction recovery of 88.2±5.8%-105±1.8%.

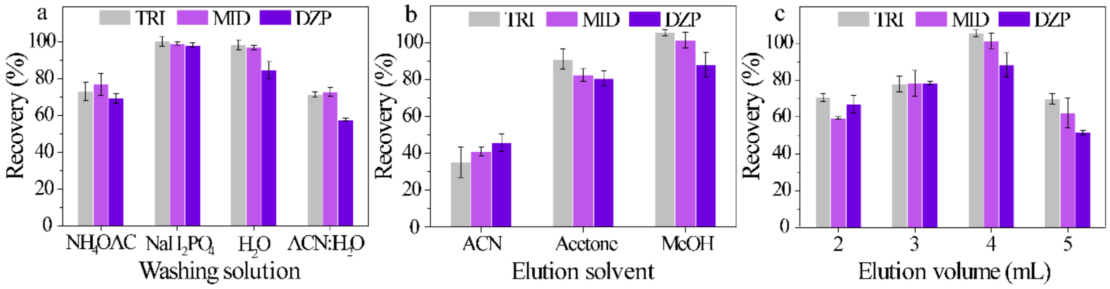


**Figure S1.** Effects of (**a-c**) washing solution, elution solvent and elution volume on the recoveries of three BZDs ($n$=3).
